# Integrated Genomic and Transcriptomic Analyses Suggest the Potential Involvement of the *COBRA* Gene Family in Heat, Drought and Combined Stress Responses in *Phoebe bournei*

**DOI:** 10.3390/biology15131084

**Published:** 2026-07-06

**Authors:** Ruobing Ying, Ronglin Liu, Yizhuo Feng, Duo Yu, Xinghao Tang, Kehui Zheng, Shijiang Cao

**Affiliations:** 1 College of Forestry, Fujian Agriculture and Forestry University, Fuzhou 350002, China; 3236805016@stu.fafu.edu.cn (R.Y.); 52404022026@fafu.edu.cn (R.L.); 12404028023@fafu.edu.cn (Y.F.); 2220428002@fafu.edu.cn (X.T.); 2 College of Agriculture, Fujian Agriculture and Forestry University, Fuzhou 350002, China; 3245002067@stu.fafu.edu.cn; 3 Fujian Academy of Forestry, Fuzhou 350012, China; 4 College of Computer and Information Sciences, Fujian Agriculture and Forestry University, Fuzhou 350002, China

**Keywords:** *Phoebe bournei*, COBRA-like proteins, combined stress, WGCNA, tertiary structure

## Abstract

The *COBRA* gene family is crucial for plant cell wall formation and stress adaptation. Although *COBRA/COBL* genes have been characterized in several plant species, their potential contribution to cell wall-mediated responses in woody plants under combined heat–drought stress remains largely unclear. In this study, eight *COBRA* genes (*PbCOBRA1*–*PbCOBRA8*) were systematically identified in *Phoebe bournei*, a highly valued timber species, and their potential stress-associated roles were investigated by integrating comparative genomic analysis, tissue expression profiling, transcriptome-based WGCNA, physiological stress indicators, qRT-PCR validation, functional enrichment analysis, and three-dimensional structure prediction. Crucially, Weighted Gene Co-expression Network Analysis (WGCNA) discovered three modules that responded to different pressures, involving a total of four *PbCOBRA* genes (*PbCOBRA1*, *PbCOBRA4*, *PbCOBRA6*, and *PbCOBRA8*). *PbCOBRA6* and *PbCOBRA8*, meanwhile, were assigned to the same module, which was uniquely associated with combined stress. With contrasting kME values, these two genes may play different roles within this module. The three-dimensional structure prediction further revealed the structural basis for *PbCOBRA* function under stress conditions. Taken together, these findings identify *PbCOBRA* members as promising stress-responsive candidate genes potentially associated with heat, drought, and combined stress responses in *P. bournei*. This study provides a theoretical basis for future functional validation and offers candidate genes for exploring cell wall-mediated stress adaptation in this valuable woody species.

## Simple Summary

*Phoebe bournei* is a valuable timber tree, but is under threat due to global warming. This study first identified 8 *COBRA* genes in *Phoebe bournei*, and has analyzed their evolutionary relationships, structural characteristics, tissue expression, and dynamic expression trends under stress conditions. Through WGCNA, which integrated transcriptome data and physiological data, we discovered four key potential candidates. Protein three-dimensional modeling further provides structural clues for understanding their potential roles under adverse conditions. These findings provide useful candidate genes and scientific clues for future studies on stress adaptation and conservation of *Phoebe bournei*.

## 1. Introduction

In the context of global warming, the frequency of extreme heat events and water deficit has continued to increase, and these two stresses often occur simultaneously or sequentially under natural conditions [1,2]. Compared to single pressure, combined stress often causes greater damages to plants, such as photosynthetic failure and low water use efficiency [3,4]. Additionally, the accumulation of reactive oxygen species (ROS) under stress, if left uncontrolled, can progressively amplify cellular damage [5]. For example, ROS-induced lipid peroxidation generates toxic substances like MDA that can further damage membrane functions and the integrity of organelles [6]. Facing these challenges, plants have evolved an antioxidant system to promptly eliminate ROS and prevent accumulation [7]. Among the important components of this system are antioxidant enzymes, including superoxide dismutase (SOD; EC 1.15.1.1), peroxidase (POD; EC 1.11.1.7), and catalase (CAT; EC 1.11.1.6). Sensing environmental stress and cellular damage, plants will activate these complex regulatory mechanisms to enhance their adaptability [8]. Therefore, analyzing these important mechanisms is of great significance for the conservation and breeding of rare plants [9]. However, studies have shown that the dynamic regulatory network under the combined stress conditions is much more complex, and it has still not been fully explored at present [8,10].

*Phoebe bournei* (Hemsl.) Yen C. Yang is an important and precious timber tree species in southern China, possessing high economic, industrial, and ecological value. Because of its durability, fine texture, and natural fragrance, it is widely used in ancient buildings, high-end furniture, and other industries, earning it the name “Golden Silk Nanmu” [11,12]. In addition, the value of *P. bournei* has been further explored in recent years. It has been proven that the essential oil from *P. bournei* exhibits inhibitory effects against *Botrytis cinerea*, expanding the potential applications of this timber tree species [13,14]. Despite the high value, *P. bournei* now faces severe threats to its habitats, due to climate changes and excessive human exploitation [15]. Previous studies have also revealed that *P. bournei* seedlings are relatively sensitive to water deficit, and drought stress can significantly affect their growth, physiological metabolism, and transcriptional regulatory networks [16,17]. As a result, investigating the physiological mechanisms of *P. bournei* under stress, and identifying the potential genetic resources for stress-resistant breeding are of great significance for the conservation and sustainable utilization of this valuable species.

The *COBRA* (*COBRA-like*, *COBL*) gene family comprises a class of plant-specific glycosylphosphatidylinositol-anchored proteins (GPI-anchored proteins), which typically possess an N-terminal signal peptide, a conserved COBRA domain, and a potential C-terminal GPI-anchor attachment site. The conserved COBRA domain, represented by the Pfam domain PF04833, is the core diagnostic feature for identifying COBRA/COBL family members. Therefore, reliable identification of COBRA proteins generally requires confirmation of this conserved domain, together with other typical structural features such as the N-terminal signal peptide and the C-terminal GPI-anchor signal. The mature proteins are generally localized to the outer side of the plasma membrane or to cell wall-associated regions [18,19,20]. According to classical studies, the *COBRA* gene family are believed to be essential for oriented cell elongation and highly anisotropic expansion, and that its function is closely associated with cellulose microfibril orientation [19,20,21]. Since the plant cell wall is not a passive static structure, but rather a dynamic interface linking cell growth, morphogenesis, and environmental perception, both drought and heat stress can induce remodeling of cell wall components, microfibril organization, mechanical properties, and associated metabolic pathways. In addition, the cell wall–plasma membrane interface is closely associated with stress perception, ROS signaling, and antioxidant regulation. Under abiotic stress, excessive ROS accumulation can induce lipid peroxidation and impair membrane and cell wall integrity, whereas antioxidant enzymes, including SOD, POD, and CAT, are activated to alleviate oxidative damage. Because COBRA/COBL proteins participate in cellulose deposition and cell wall organization, they may be linked to the ROS–antioxidant enzyme (AOE) nexus by maintaining cell wall integrity and influencing stress-responsive signaling processes [22,23,24]. Consequently, the COBRA gene family has attracted considerable attention in the context of abiotic stress responses. In *Arabidopsis thaliana* and *Oryza sativa*, the specific functions of individual *COBRA* genes have been extensively studied. *OsBC1* and *AtCOBL4*, for example, have been determined to be involved in the biosynthesis of secondary cell wall cellulose and to control mechanical strength [25,26]. Meanwhile, *AtCOBL9* was found to be essential for the elongation of root hairs in *Arabidopsis thaliana*, reflecting the role of the *COBRA* gene family in highly polarized dynamic cell wall remodeling [26]. Apart from model plants, extensive studies on the *COBRA* gene family have also been conducted in diverse plant species. In wheat, *TaCOBL6A2* has been demonstrated to improve thermotolerance, and other *TaCOBL* members also respond markedly to drought treatment [27,28]. In addition, *COBL* family members in rapeseed, sorghum, and cotton show significant expression changes under drought, PEG, or salt stress, and silencing of *GhCOBL22* reduces drought tolerance in cotton [29,30,31]. However, the *COBRA* gene family in *P. bournei* has not yet been systematically identified to date, and its potential involvement in the responses to heat, drought, and combined heat-drought stress remain unclear as well.

This study aimed to identify *COBRA* genes in *P. bournei* and to comprehensively analyze their evolutionary relationships, structural characteristics, tissue-specific expression patterns, and stress-responsive expression profiles. Combined with RT-qPCR analysis, it revealed *PbCOBRA* genes dynamic expression trends under three treatments. Importantly, this study integrated transcriptome data with physiological data for WGCNA to explore potential gene resources in the *COBRA* gene family. Through GO and KEGG enrichment, the complex regulatory mechanisms of *P. bournei* under stress were explored. Additionally, the three-dimensional modeling of four key PbCOBRA proteins further revealed the structural basis of their functions under environmental pressures. These findings provide a theoretical basis for understanding the potential involvement of *PbCOBRA* genes in stress-responsive regulatory networks of *P. bournei* and offer promising candidate genes for future functional studies of cell wall-mediated adaptation under abiotic stress.

## 2. Materials and Methods

### 2.1. Recognition of PbCOBRA Gene Family

The complete protein and genome sequences, along with the GFF3 file of *P. bournei* were from the CNGB database (https://db.cngb.org/search/project/CNP0002030/, accessed on 19 March 2026), while those of *Arabidopsis thaliana* and *Oryza sativa* were from NCBI Database and RGAP (https://rice.uga.edu, accessed on 19 March 2026) respectively. Using HMMER software (v3.3.2), the conserved domain of COBRA family (PF04833) was screened with an E-value < 1 × 10^−5^. Then, we performed BLASTP searches, which used *AtCOBL* sequences as queries. To ensure accuracy, all gene candidates were manually cross-checked against the NCBI Conserved Domain Database (CDD) (https://www.ncbi.nlm.nih.gov/Structure/cdd/cdd.shtml, accessed on 19 March 2026) and the Pfam database (http://pfam.xfam.org/, accessed on 19 March 2026). Physicochemical properties and subcellular localization were analyzed using the ExPASy ProtParam tool (https://web.expasy.org/protparam/, accessed on 19 March 2026), and WoLF PSORT (https://wolfpsort.hgc.jp/, accessed on 19 March 2026) respectively.

### 2.2. Chromosomal Location and Structure Analysis

We used genomic annotation of *P. bournei* to determine the chromosomal locations of each *PbCOBRA* family member, and the results were then visualized through TBtools-II (v2.330) [32]. As for structural analysis, the gene constitutions were displayed employing the TBtools-II (v2.330) with GFF annotation file of the *P. bournei* genome. To view protein structure, domains were checked by the NCBI Batch CD-Search tool (https://www.ncbi.nlm.nih.gov/Structure/bwrpsb/bwrpsb.cgi, accessed on 19 March 2026), while the conserved motifs were probed with the MEME Suite (v5.3.3) (https://meme-suite.org/meme/tools/meme, accessed on 19 March 2026). Conserved motifs were identified with a maximum set to ten, thereby balancing the capture of major structural features against the generation of superfluous minor patterns. These motifs and domain distributions were then structured and visualized in TBtools-II (v2.330). For an initial assessment of regulatory elements, 2000 bp promoter regions of the *COBRA* genes were extracted, and next screened using the PlantCARE database (http://bioinformatics.psb.ugent.be/webtools/plantcare/html/, accessed on 20 March 2026).

### 2.3. Phylogenetic and Collinearity Analysis

The protein sequences of *Populus trichocarpa* (Pop_tri_v4), *Arabidopsis thaliana* (TAIR10), and *Oryza sativa* (IRGSP-1.0) were retrieved from the EnsemblPlants database (https://plants.ensembl.org, accessed on 2 April 2026), NCBI Database, and RGAP (https://rice.uga.edu, accessed on 19 March 2026) respectively. Multiple sequence alignment was carried out using ClustalW implemented in MEGA 12.0 with default parameters. Before tree construction, we determined the best-fit substitution model (LG + G) based on the lowest Bayesian Information Criterion (BIC) score using the model selection tool in MEGA 12.0. Subsequently, a phylogenetic tree was constructed using the Maximum Likelihood (ML) method, with branch support evaluated by 1000 bootstrap replicates [33]. Finally, the tree was beautified in iTOL (https://itol.embl.de/, accessed on 2 April 2026). Collinearity analysis was conducted using the One-Click MCScanX function in TBtools-II (v2.330) and visualized with the Dual Synteny Plotter plugin [32,34].

### 2.4. Tissue-Level Expression Profiling

The expression profiles of the *PbCOBRA* gene family were analyzed based on transcriptome data from various tissues of *P. bournei*. A clustered heatmap was generated using the Heatmap module in TBtools-II (v2.330), with expression values displayed as log_2_ (FPKM + 1) [32].

### 2.5. Three-Dimensional Structure Modeling

Template search was performed against the SWISS-MODEL template library (https://swissmodel.expasy.org, accessed on 10 April 2026) [35]. The most suitable templates were selected based on Global Model Quality Estimate (GMQE) and sequence identity. For all four PbCOBRA proteins, the selected templates showed high sequence identities (>80%) and favorable GMQE scores (>0.80), ensuring the reliability of the resulting 3D models. The N-glycosylation site was predicted in NetNGlyc 1.0 (https://services.healthtech.dtu.dk/services/NetNGlyc-1.0/, accessed on 4 May 2026), and only sites rated as “++” or higher were retained. The GPI anchor site was forecast by NetGPI 1.1 (https://services.healthtech.dtu.dk/services/NetGPI-1.1/, accessed on 4 May 2026). The final results were visualized in PyMOL version 3.0.3 (Schrödinger, LLC, New York, NY, USA).

### 2.6. Plant Materials and Treatment

The one-year-old seedlings of *P. bournei* used in this study were sourced from Fujian Academy of Forestry. These well-growing and similar plants were then placed in an artificial incubator for adaptation and cultivation for about 20 days. The cultivation parameters were set as follows: watering twice daily at 07:00 and 17:00, a light intensity of 600 μmol m^−2^ s^−1^, a photoperiod of 14 h light/10 h dark, a constant temperature of 25 °C, and relative humidity ranging from 60% to 70%. Following the recovery phase, uniform and healthy *P. bournei* seedlings were equally divided into three groups and exposed to three treatments: high temperature (H, 40 °C), drought (D, 10% PEG6000, CP grade, Sinopharm Chemical Reagent Co., Ltd., Shanghai, China), and combined stress (HD). The concentration of 10% PEG6000 was selected because previous studies have shown that 10% PEG6000 can effectively simulate drought conditions (−0.15 MPa) and induce physiological responses of *P. bournei* [17,36]. All seedlings were grown in soil. Under the high temperature treatment, the soil was kept consistently moist by watering twice daily, while drought and combined stress were applied by irrigating with 10% PEG6000 solution. Starting at 9:00 a.m., leaf samples were collected at five time points: 0 h (CK), 1 h, 12 h, 24 h, and 48 h post-treatment. At each time point, mature leaves were harvested from all three treatment groups. Leaves from three seedlings were pooled as one biological replicate, and three biological replicates were prepared per treatment per time point. Physiological indicators such as RWC and *F_v_*/*F_m_* were measured using fresh leaves, while the samples for RNA-seq were snap-frozen in liquid nitrogen and stored at −80 °C until sequencing, which was performed by Fuzhou Qingbaiwang Biotechnology Co., Ltd (Fuzhou, Fujian, China).

### 2.7. cDNA Library Construction and Sequencing

Transcriptome sequencing was performed on samples from the control (CK) and the time points showing significant responses (1 h, 12 h, 24 h, and 48 h) in each treatment group. Three biological replicates were included per treatment, resulting in a total of 45 samples. Total RNA was extracted from the 45 *P. bournei* leaf samples using the RNAprep Pure Plant Plus Kit (Polysaccharide & Polyphenolic-rich) (Tiangen Biotech Co., Ltd., Beijing, China). RNA purity was assessed using a NanoDrop 2000 spectrophotometer (Thermo Fisher Scientific, Waltham, MA, USA). Additionally, RNA integrity was accurately evaluated using the Agilent 2100 Bioanalyzer system (Agilent Technologies, Santa Clara, CA, USA). Only high-quality RNA samples (OD260/280 = 1.8~2.2, OD260/230 ≥ 2.0, and RIN ≥ 6.5) were utilized for cDNA library construction. The RNA-seq transcriptome libraries were prepared following standard procedures using 1 μg of total RNA. Briefly, messenger RNA was isolated via polyA selection using oligo(dT) beads and subsequently fragmented. cDNA synthesis, end repair, A-base addition, and the ligation of indexed adaptors were then performed. The libraries were size-selected for cDNA target fragments of 200–300 bp on 2% Low Range Ultra Agarose, followed by PCR amplification using Phusion DNA polymerase for 15 cycles. After quantification, the final libraries were sequenced on an MGI-T7 sequencing platform (Shanghai BIOZERON Biotech. Co., Ltd., Shanghai, China) using the paired-end 150 bp (PE150) mode. For basic quality control, raw paired-end sequencing data were subjected to adapter trimming and quality filtering using Trimmomatic (version 0.39). Read alignment quality was evaluated using RSeQC (version 2.6.4).The clean reads were then mapped to the *P. bournei* reference genome using HISAT2 (v2.2.1), and transcript assembly and expression quantification were performed using StringTie (v2.1.7). The raw RNA-seq data generated in this study have been deposited in the *Phoebe bournei* Genome Database (https://pbgenome.org.cn/).

### 2.8. Weighted Gene Co-Expression Network Analysis (WGCNA)

Weighted gene co-expression network analysis (WGCNA) was performed using the WGCNA Shiny plugin in TBtools-II (v2.453) [32,37]. To ensure network robustness, genes were filtered based on median absolute deviation (MAD), and the top 9000 highly variable genes were retained. The raw RNA-seq read counts were normalized to Counts Per Million (CPM) followed by a $log_{2}(CPM+1)$ transformation. A co-expression network was constructed using a soft-thresholding power of 8. Module–trait associations were then evaluated between module eigengenes and physiological parameters, including SOD, POD, CAT, MDA, RWC, SP, and $F_{v}/F_{m}$. Module membership (kME) values were calculated to assess the correlation between individual gene expression profiles and module eigengenes.

### 2.9. Measurement of Physiological Indicators

Mature leaves of *P. bournei* from each treatment and the control group were collected and immediately used for physiological measurements. Superoxide dismutase (SOD; EC 1.15.1.1) activity was determined by the nitroblue tetrazolium (NBT) photoreduction method [38]. Peroxidase (POD; EC 1.11.1.7) activity was measured using the guaiacol colorimetric method [39]. Catalase (CAT; EC 1.11.1.6) activity was assayed by ultraviolet absorption [39]. Malondialdehyde (MDA) content was determined by the thiobarbituric acid (TBA) colorimetric method [40]. Soluble protein (SP) content was measured using the Coomassie brilliant blue G-250 staining method [41]. Relative water content (RWC) of leaves was determined using the gravimetric method [42]. Chlorophyll fluorescence parameters were measured using a portable pulse-amplitude-modulated (PAM) fluorometer, and the maximum quantum efficiency of photosystem II (*F_v_*/*F_m_*) was calculated [43]. Additionally, plant leaves were photographed at each sampling time point.

### 2.10. GO and KEGG Enrichment Analysis

Based on the WGCNA results, all genes assigned to the turquoise module were subjected to GO and KEGG enrichment analyses using the *P. bournei* Genome Database (https://pbgenome.org.cn/, accessed on 7 April 2026), and TBtools-II (v2.330) [32]. For GO enrichment analysis, the Benjamini–Hochberg method was applied for *p*-value adjustment, with a significance threshold of *p* < 0.05 and Q < 0.05. The gene set size was restricted to a minimum of 15 and a maximum of 200 genes. For KEGG pathway enrichment analysis, significantly enriched pathways were identified using a threshold of *p* < 0.05 and Q < 0.05.

### 2.11. qRT-PCR Analysis

To comprehensively explore the stress-responsive patterns of all identified *PbCOBRA* family members, an optimized qRT-PCR analysis was performed using an independent batch of *P. bournei* seedlings, which experienced the same treatment as mentioned in Section 2.6. Based on general plant physiological principles, the sampling time points were adjusted to enhance temporal resolution. Specifically, the late-stage 48 h point was removed, and a 6 h point was added to better capture the critical transition phase of the stress response, resulting in a refined series: 0, 1, 6, 12, and 24 h. Total RNA was extracted from mature leaves using a specialized plant RNA extraction kit (TSP412, Tsingke, Beijing, China). RNA purity and concentration were verified using an Epoch microplate spectrophotometer (BioTek, Winooski, VT, USA). First-strand cDNA was synthesized from 2 μg of total RNA using the SynScript^®^ III RT SuperMix (DLR101, Tsingke, Beijing, China) with integrated genomic DNA removal. The qRT-PCR assays were performed on an ABI QuantStudio StepOne Plus system using the ArtiCanCEO SYBR qPCR Mix (DLQ101, Tsingke, Beijing, China). A 20 μL qPCR mix was assembled containing 10 μL 2× SYBR Mix, 0.8 μL each primer (10 μM), 1 μL diluted cDNA (1:4), and 7.4 μL water. The amplification program consisted of 95 °C for 5 min, followed by 40 cycles of 95 °C for 15 s, 60 °C for 20 s, and 72 °C for 20 s. A dual-reference gene normalization strategy was adopted. The geometric mean of the threshold cycle (Ct) values from PbEF1α (GenBank: KX682032) and PbCBP20 was used for normalization. Relative expression levels of all eight *PbCOBRA* genes were calculated via the 2^−ΔΔCt^ method. Statistical significance was determined by one-way ANOVA followed by Dunnett’s T3 post-hoc test (*p* < 0.05) using GraphPad Prism software (v.10.1.2). The primer sequences employed in this study are shown in Table 1.

## 3. Results

### 3.1. Identification and Physicochemical Properties of PbCOBRA Gene Family

Validated by BLAST and HMM methods, followed by manual verification, a total of 8 *PbCOBRA* genes were discovered from the whole genome of *P. bournei*, and they were renamed as *PbCOBRA1* to *PbCOBRA8* (Table 2). Through online tools including ExPASy ProtParam and WoLF PSORT, the basic properties of PbCOBRA proteins were obtained, as shown in Table 2 as well. The amino acid lengths of eight PbCOBRA proteins ranged from 411 aa (PbCOBRA3) to 661 aa (PbCOBRA5), while the molecular weights ranged from 46.379 kDa (PbCOBRA3) to 73.773 kDa (PbCOBRA5). The theoretical isoelectric points (pI) varied from 5.5 (PbCOBRA4) to 9.12 (PbCOBRA1), including 3 acidic proteins (pI < 6.5) and 5 basic proteins (pI > 7.5), indicating distinct divergence in charge properties among family members. Instability index analysis revealed that all members were stable proteins (instability index < 40) except PbCOBRA3 (44.11) and PbCOBRA8 (44.67). The GRAVY values of all PbCOBRA proteins were negative (−0.331 to−0.053), confirming that they were hydrophilic proteins. The aliphatic indexes ranged from 68.52 to 80.55. PbCOBRA4 (80.55), notably, exhibited relatively high aliphatic indexes. As for subcellular localization prediction, PbCOBRA proteins were predominantly located in the plasma membrane (4), vacuole (1), cytoplasm (1), endoplasmic reticulum (1), and chloroplast (1). The high proportion of plasma membrane-localized members is consistent with the basic functional characteristics of COBRA proteins involved in cell wall biosynthesis and cellulose deposition.

### 3.2. Phylogenetic Analysis of the PbCOBRA Gene Family

**Figure 1:**
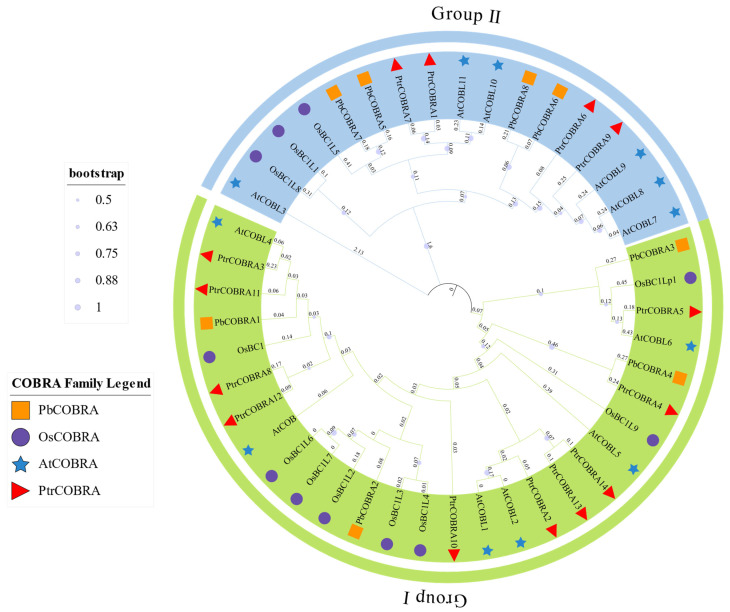
The phylogenetic tree of COBRA proteins in four species (8 proteins from *P. bournei*, 12 from *Arabidopsis thaliana*, 11 from *Oryza sativa*, and 14 from *Populus trichocarpa*). Different colors and shapes were represented different subgroups and COBRA families, respectively.

The multispecies phylogenetic tree explored the evolutionary process. Protein sequences of the COBRA family in *Populus trichocarpa*, *Oryza sativa*, *Arabidopsis thaliana* and *P. bournei* were used to construct the tree by the Maximum Likelihood (ML) method with 1000 bootstrap replicates. According to Figure 1, all the COBRA proteins were divided into two subfamilies (Group I-II). The PbCOBRA members were distributed evenly in the two groups. In Group II, PbCOBRA5-8 formed a subclade with AtCOBL9, which is known to function in highly polarized dynamic cell wall remodeling [26]. In comparison, PbCOBRA1-4 were clustered with well-known stress-resistant AtCOBL4 and OsBC1 in Group I, both of which have been proven to play a vital role in secondary cell wall formation and mechanical strength, suggesting that these PbCOBRA proteins may have potential roles in cell wall-related stress responses in *P. bournei* [25,26]. The phylogenetic grouping suggested possible conservation and divergence among PbCOBRA members; therefore, conserved motifs, conserved domains, and exon–intron structures were further analyzed to determine whether these evolutionary relationships were supported by structural features.

### 3.3. Structural Characterization of the PbCOBRA Gene Family

**Figure 2:**
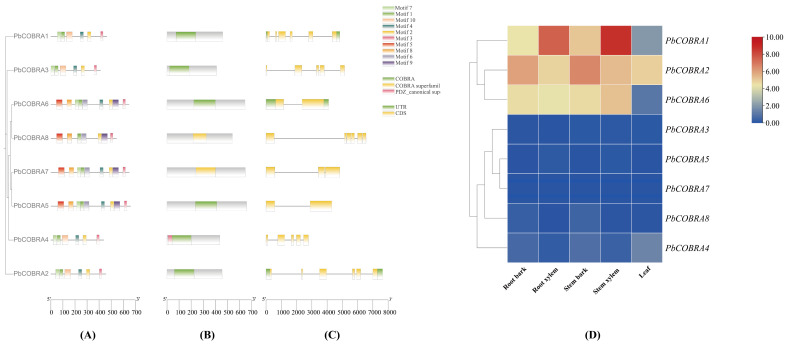
(**A**) Conserved motifs of PbCOBRA proteins. The 10 conserved motifs are represented by rectangles with different colors and numbers. (**B**) Conserved domains of PbCOBRA proteins. (**C**) Schematic diagram of *PbCOBRA* gene structures. Green and yellow rectangles represent coding sequences (CDS) and untranslated regions (UTR), respectively. Black lines indicate introns. (**D**) Expression profiles of *PbCOBRA* genes in different tissues of *P. bournei*. The color gradient represents the gene expression level, with red indicating high expression and blue indicating low expression. Tissues include root bark, root xylem, stem bark, stem xylem, and leaf.

To further investigate the structure features and evolutionary relationships of PbCOBRA proteins, conserved motifs were predicted using the MEME online tool, and conserved domains were identified by searching against the NCBI-CDD databases. Meanwhile, the exon–intron structures of *PbCOBRA* genes were analyzed based on the *P. bournei* genome annotation (Figure 2A–C).

Ten conserved motifs (motif 1–10) were identified in PbCOBRA proteins via MEME analysis. As shown in Figure 2A, motif 1, motif 2, and motif 3 were present in all members, indicating that these motifs likely represent the core regions of this family and are essential for maintaining the basic functions. Notably, PbCOBRA5, 6, and 7 each contained 9 identical motifs with the most complete composition, suggesting structural conservation and similar functions in these proteins. In contrast, PbCOBRA1, 2, 3, and 4 each contained only 6 conserved motifs with identical composition among the four members, implying that these four members may have undergone motif loss or simplification during evolution, potentially leading to functional divergence.

Comparison against NCBI-CDD databases (Figure 2B) revealed that PbCOBRA1, 2, 3, 4, 5, and 6 contained the typical COBRA family domain, which was the defining feature of the COBRA family, while PbCOBRA7 and 8 contained the COBRA superfamily domain. In addition, PbCOBRA4 harbored a PDZ_canonical superfamily domain.

The exon–intron structures of *PbCOBRA* genes were further analyzed based on the *P. bournei* genome annotation. As illustrated in Figure 2C, *PbCOBRA* genes exhibited obvious structural diversity. The number of exons varied from 2 to 6 among different *PbCOBRA* genes. Regarding UTR distribution, several genes (e.g., *PbCOBRA1*, *PbCOBRA2*, and *PbCOBRA6*) contained both 5′ UTR and 3′ UTR, whereas UTR structures were incomplete or absent in other members. Structural diversity is a common characteristic of functional divergence among gene family members. Differences in exon number and UTR distribution among *PbCOBRA* genes imply variations in transcriptional regulation and translation efficiency, which may further influence their expression patterns and functions across diverse tissues or under stress conditions.

### 3.4. Expression Pattern of PbCOBRA Family Members in Different Tissues

Through *P. bournei* transcriptome data, the transcript profiles of eight *PbCOBRA* genes in multiple tissues were analyzed. The results showcased distinct tissue-specific expression characteristics among the different members (Figure 2D). Among them, *PbCOBRA1* exhibited the highest expression level in the xylem of both roots and stems, while being barely expressed in leaves. This implied that it may contribute to the development of xylem or secondary cell wall synthesis. *PbCOBRA2* was broadly expressed across all examined tissues at moderate levels, displaying a constitutive expression pattern. *PbCOBRA6* showed moderate expression in roots and stems but was nearly undetectable in leaves. Notably, *PbCOBRA3*, *PbCOBRA4*, *PbCOBRA5*, *PbCOBRA7*, and *PbCOBRA8* showed extremely low expression levels across all tested tissues, being almost undetectable. This indicates that these genes may be subject to strict spatiotemporally regulated expression, and their functions might be induced only by specific developmental stages or environmental stress signals. These tissue-specific expression patterns provided a baseline view of *PbCOBRA* gene activity under normal conditions. 

### 3.5. Chromosomal Mapping and Synteny Analysis of COBRA Genes in P. bournei, A. thaliana and O. sativa

**Figure 3:**
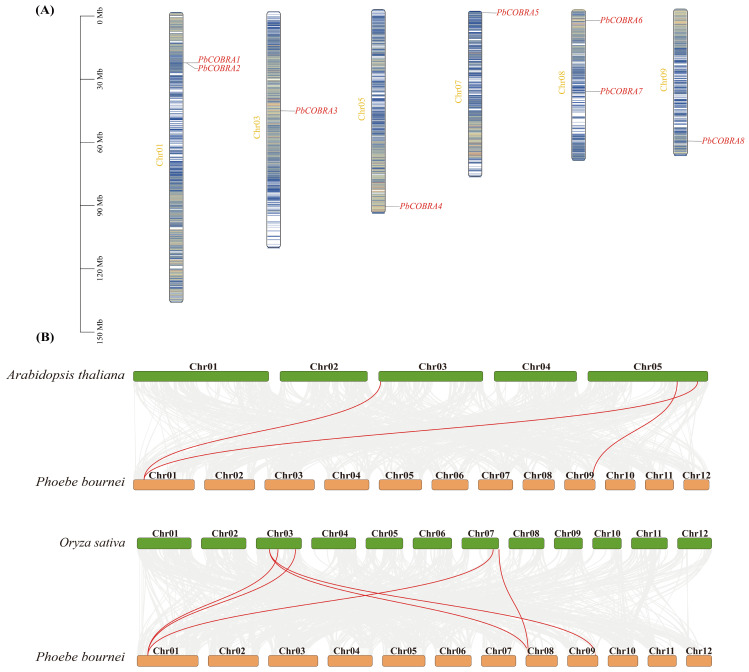
(**A**) Overview of PbCOBRA gene mapping. The colors on the chromosomes represent gene density, where blue, yellow, and white regions indicate low, high, and zero gene density, respectively. (**B**) Homology analysis was conducted on the COBRA genes in *P. bournei*, *A. thaliana*, and *O. sativa*. The gray lines represented the alignment regions between the paired genomes, while the red lines indicated the homologous PbCOBRA gene pairs.

With *P. bournei* genomic annotation, a visualized distribution map was constructed to view the precise chromosomal localization of *PbCOBRA* genes (Figure 3A). The analysis revealed that 8 *PbCOBRA* genes distributed unevenly across 6 chromosomes. To be specific, Chr01 and Chr08 contained two *PbCOBRA* genes, whereas other chromosome possessed only one *PbCOBRA* gene. It was worth noting that most *PbCOBRA* members distributed separately, in addition to a gene cluster consisting of *PbCOBRA1* and *PbCOBRA2* was discovered on Chr01. These findings suggested that the genomic expansion of the *PbCOBRA* gene family was restricted during evolution, likely subjected to strict evolutionary constraints against gene redundancy.

Collinear analysis was next performed to further study the expansion mechanism of *PbCOBRA* gene family (Figure 3B). The interspecific analysis was conducted using the sequence files from *P. bournei*, *A. thaliana* and *O. sativa*. Since *P. bournei* and *A. thaliana* are both dicotyledons, their evolutionary relationships are expected to be closer. However, results showed that there were three orthologous gene pairs between *AtCOBRA* and *PbCOBRA*, while six pairs were found between *OsCOBRA* and *PbCOBRA*. The discrepancy in the number of collinear pairs between the two species may be attributed to the specific evolutionary history of the *COBRA* family. It is possible that the genome of *O. sativa* experienced specific whole-genome duplication (WGD) events, which might have led to the retention of more *COBRA* collinear pairs compared to *A. thaliana* [44].

Furthermore, no intra-species collinearity was detected within the *PbCOBRA* gene family, indicating that its expansion mechanism during evolution is highly conservative, which is consistent with the previous analyses.

### 3.6. Cis-Acting Element Analysis of PbCOBRA Genes

**Figure 4:**
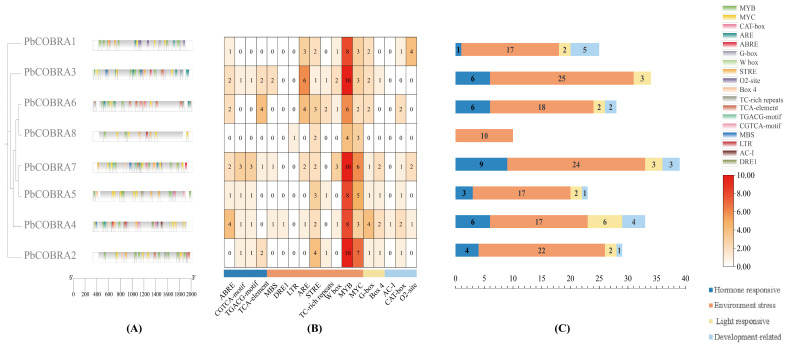
Overview of cis-acting elements in the *PbCOBRA* genes. (**A**) The distribution of elements in the 2000 bp upstream sequences of *PbCOBRA* genes. (**B**) The abundance of 18 element types. (**C**) Quantifies elements by four functional categories.

Extracting the 2000 bp promoter region upstream of *PbCOBRA* genes, we analyzed the cis-acting element of this family by using the PlantCARE database. The results identified 18 types of elements in total, and they were classified into four functional groups: hormone-responsive, environmental stress-responsive, light-responsive, and development-related elements (Figure 4). The heatmap showed that the MYB element exhibited the highest abundance in all *PbCOBRA* genes (mostly 10 copies). Meanwhile, environmental stress-related elements such as STRE, ARE, and TC-rich repeats were widely distributed, whereas hormone-responsive elements (e.g., ABRE, CGTCA-motif) and development-related elements (e.g., CAT-box) showed obvious divergence in abundance among different family members.

Functional classification statistics revealed that environmental stress-responsive elements were absolutely dominant in all *PbCOBRA* genes (ranging from 10 to 25 copies), indicating that this family has great potential in plant abiotic stress responses. The number of hormone-responsive elements varied the most (0 to 9 copies), reflecting functional divergence of family members in hormonal signaling regulation. Light-responsive and development-related elements were less abundant and enriched only in certain genes. For instance, *PbCOBRA1* contained the most development-related elements (5 copies), *PbCOBRA7* harbored the highest number of hormone-responsive elements (9 copies), and both *PbCOBRA7* and *PbCOBRA3* were highly involved in the coordinated regulation of stress and hormone signaling. Certain members were also found to lack specific element categories. The *PbCOBRA8* only had environmental stress-responsive elements. By contrast, *PbCOBRA3* lacked development-related elements. These findings revealed that different family members may possess distinct specialized functions, which could provide ro. To further investigate whether these genes are associated with abiotic stress responses, transcriptome-based WGCNA was performed under heat, drought, and combined stress conditions.

### 3.7. Weighted Gene Co-Expression Network Analysis (WGCNA) and Identification of Stress-Associated Modules

**Figure 5:**
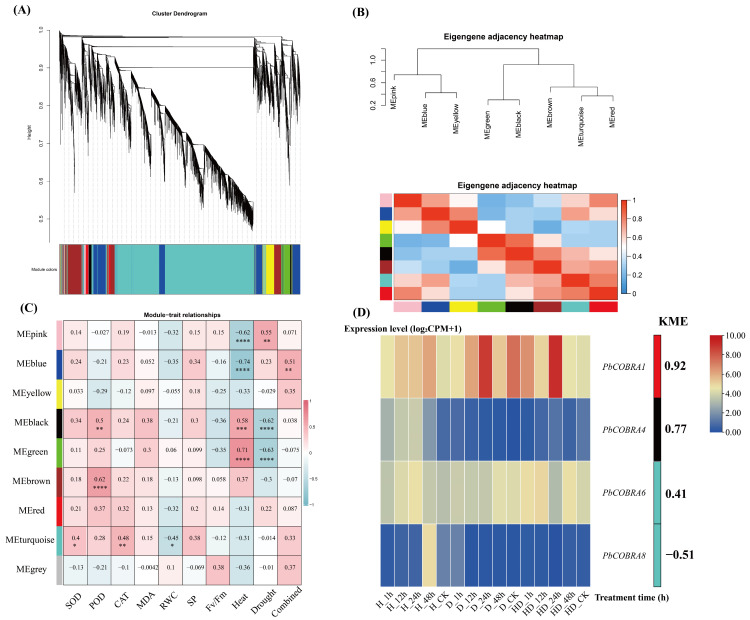
Weighted gene co-expression network analysis (WGCNA) of *P. bournei* under various abiotic stresses. (**A**) Gene dendrogram and module assignment. (**B**) Eigengene adjacency heatmap showing the correlations among different modules. (**C**) Module-trait relationship heatmap showing the correlation coefficients between modules, physiological indicators, and stress treatments. (**D**) Expression patterns of four *PbCOBRA* genes at different time points under single heat, drought, and combined stresses. The numbers on the right represent the module membership (kME) values of the genes in their respective modules. In panel (**C**), asterisks indicate statistical significance based on the Benjamini–Hochberg (BH) adjusted *p*-values: * *p* < 0.05, ** *p* < 0.01, *** *p* < 0.001, **** *p* < 0.0001.

To deeply explore the underlying regulatory mechanism, 45 transcriptomic samples spanning a longer time series were utilized for co-expression network construction (Figure 5). And physiological indicators centering on the antioxidant system (SOD, POD, CAT) and lipid peroxidation (MDA), complemented by water status (RWC), soluble protein content (SP), and photosynthetic efficiency (*F_v_*/*F_m_*) were selected to conduct module-trait relationship analysis. Nine distinct modules were identified, and there were unique features among these modules. As shown in Figure 5, some modules were significantly related to stress, such as pink, blue, black, and green. However, their correlations with the oxidative system varied considerably. Genes in the black module, for example, showed expression patterns significantly correlated with SOD (r = 0.34), CAT (r = 0.24), POD (r = 0.5) activities and MDA content (r = 0.38), as well as high correlation with heat- response (r = 0.58). By contrast, the green module exhibited high potential in heat response (r = 0.71), but showed little correlation with the antioxidant enzymes. Another instance is the pink module, which significantly responded to drought (r = 0.55), yet was weakly correlated with antioxidant enzyme activities. This implied that genes in the pink module may participate in drought response via other pathways, while black module might be involved in regulating the antioxidant system to improve plant stress-resistance. The nine different modules also showed a specialization among the three treatments. To be specific, though the black module was highly responsive to high temperature, it was significantly negatively correlated to drought. The green part demonstrated the similar trend with the black module. In addition, modules such as blue, yellow, turquoise, and grey showed no significant positive association with single stress, but were markedly induced by combined stress. The turquoise module, of note, contained the largest number of genes (5364 genes), and illustrated relatively high and significant positive correlation with SOD (r = 0.4), and CAT (r = 0.48), activities while exhibiting negative correlation with RWC (r = −0.45). This highlighted the relevance of the turquoise module and implied that genes in this module may represent important candidates for further investigation of combined stress responses.

To further investigate the specific roles of the *PbCOBRA* gene family within this complex stress-response network, we tracked the distribution of all eight *PbCOBRA* members in the co-expression network. The results demonstrated that four *PbCOBRA* genes were assigned to three co-expression modules, with two members (*PbCOBRA6* and *PbCOBRA8*) sharing the turquoise module. The transcriptomic expression profiles of the four candidate genes at each stress time point are shown in Figure 5D, together with their module membership (kME) values. Interestingly, the *PbCOBRA1* was assigned to red module with a considerably high kME (0.92), which indicated that the *PbCOBRA1* might play a crucial role in the red module. The transcriptome data also depicted that its transcript abundance was relatively high in *P. bournei* under combined and single drought stress, implying its significance in stress responses. *PbCOBRA4* was co-expressed with genes in the black module with a comparatively high kME (0.77), suggesting a potentially important role in this module. Prominently, *PbCOBRA6* and *PbCOBRA8* were co-assigned to the turquoise module with 0.41 and −0.51 kME respectively. Although the kME values of *PbCOBRA6* and *PbCOBRA8* were moderate, their membership in the turquoise module, which was specifically related to combined stress, suggested their possible contribution to combined stress adaptation. Since the turquoise module was specifically associated with combined stress and contained two PbCOBRA candidates, this module was selected for subsequent functional enrichment analysis to explore the biological processes and pathways potentially linked to combined stress adaptation.

### 3.8. Functional Enrichment Analysis

**Figure 6:**
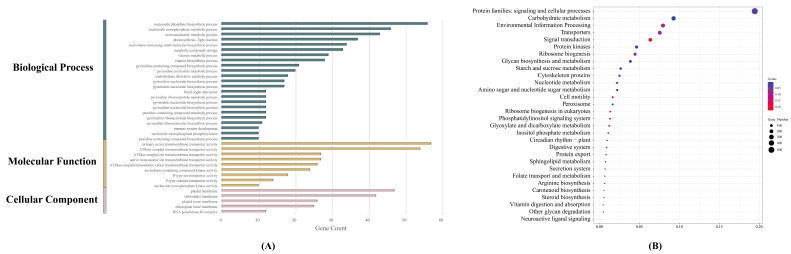
GO and KEGG functional enrichment analysis. (**A**) GO enrichment analysis. The different categories were represented by different colors. (**B**) KEGG enrichment analysis. Blue color indicates smaller *Q*-values, while red color indicates larger *Q*-values.

Given that the turquoise module in WGCNA contained the most genes, and showed specificity of combined stress, we extracted all the genes in the module to conduct GO and KEGG enrichment analysis in order to further reveal the molecular mechanism of *P. bournei* tolerance to combined stress. The enriched GO entries can be classified into three categories: Biological Process, Cellular Component, and Molecular Function. The GO enrichment analysis (Figure 6A) also provided molecular evidence for the discoveries in the WGCNA analysis. In the Molecular Function category, genes were highly enriched in “primary active transmembrane transporter activity” and “ion transmembrane transporter activity”. This supported the findings in the previous analysis that the genes of this module were negatively correlated with RWC and positively correlated with SP. In the Biological Process and Cellular Component categories, the enriched terms were predominantly associated with “monosaccharide metabolic process”, “photosynthesis, light reaction”, and “chloroplast/plastid membrane”. The KEGG pathway enrichment analysis (Figure 6B) further explained the systemic regulatory network. The enrichment of “Signal transduction” and “Protein kinases” implied that genes in this module can actively respond to combined stress. Moreover, the “Peroxisome” pathway was highly enriched, which was the primary cellular compartment for Catalase (CAT) activity, further supporting the high correlation between turquoise module and CAT activity (r = 0.48) in WGCNA analysis. Most importantly, pathways essential for cell wall integrity, including “Carbohydrate metabolism”, “Glycan biosynthesis and metabolism”, “Starch and sucrose metabolism”, and “Cytoskeleton proteins”, were prominently enriched. Since glycans and carbohydrates are the fundamental building blocks of the plant cell wall, the integration of *PbCOBRA6* and *PbCOBRA8* within this module were hypothesized to play roles in the adaptation of *P. bournei* to complex abiotic stresses.

### 3.9. Tertiary Structure of the Key Proteins

Based on the WGCNA results, only four genes (*PbCOBRA1*, *PbCOBRA4*, *PbCOBRA6*, and *PbCOBRA8*) were assigned to specific co-expression modules, while the remaining members were not assigned to any modules. Therefore, we selected these four key candidates for subsequent three-dimensional (3D) structure modeling using the SWISS-MODEL website The sequence identity of all the templates with the target protein was above 80%, ensuring the accuracy of the models. Then, the predictions of N-glycosylation site and GPI-anchor site were achieved by NetNGlyc 1.0 and NetGPI 1.1 respectively.

**Figure 7:**
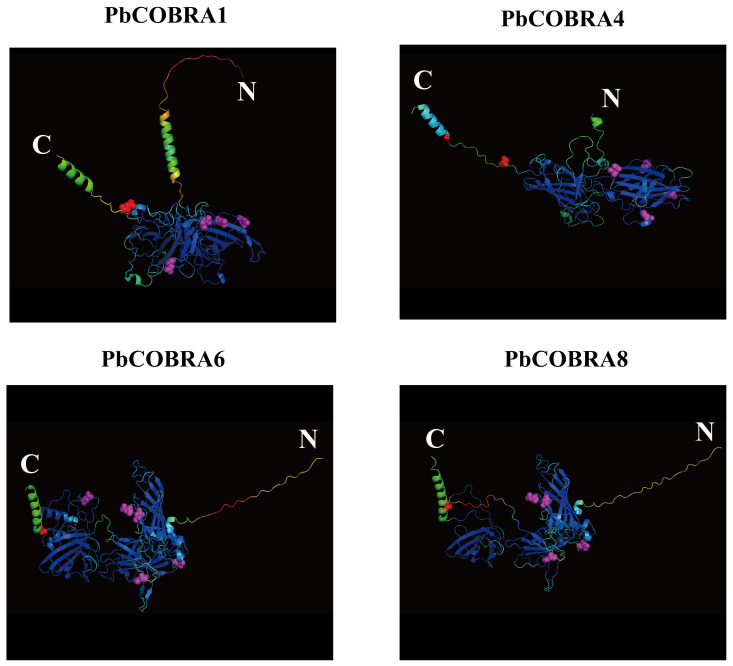
Three-dimensional structure prediction of four PbCOBRA proteins, including PbCOBRA1, PbCOBRA4, PbCOBRA6, and PbCOBRA8. The protein was colored based on the degree of confidence. Blue indicates high confidence, while red indicates the opposite. The pink spheres represented N-glycosylation sites, and red spheres represented GPI-anchor attachment sites. N and C respectively represent the N-terminal and C-terminal of the protein.

As illustrated in Figure 7, all four proteins displayed the typical conserved topological architecture of the COBRA-like family. The modeling confidence of the central core region marked in dark blue was extremely high, with a tight fold and mainly composed of stable β-sheet structures. This highly conserved rigid core visually constitutes the main structure of the protein. In contrast, the N-terminal and C-terminal extension regions, which were marked in cyan, green, yellow or red to indicate lower structural confidence, showed significant intrinsic flexibility. Moreover, the N-terminal usually formed a loose coiling extending outward from the core, except for PbCOBRA1 that possessed an α-helix in the N-terminal. The C-terminal extension region of all four proteins, conversely, contained a clear short α-helix structure. It is worth noting that multiple N-glycosylation sites represented by pink spheres were abundantly and specifically concentrated on the surface of the rigid β-sheet region of the core, whereas the flexible terminal tail chains were not glycosylated. Additionally, the GPI anchor modification site marked by a single red sphere was always located within the flexible C-terminal extension region, close to the upstream position of the C-terminal α-helix. These specific architectural features provide structural clues for understanding the potential functions of PbCOBRA proteins.

### 3.10. Quantitative Real-Time Polymerase Chain Reaction Analysis

In the preceding transcriptomic analysis, gene expression was quantified by the method of $log_{2}(CPM+1)$, which reflected the overall proportion of a given transcript in the total mRNA pool. Although the heatmap in Figure 5D had shown that certain members like *PbCOBRA1* maintained relatively high signal intensities under severe stress compared to others, highlighting their overall importance in the genome, this global perspective cannot capture the dynamic and temporal expression patterns of individual genes in response to stress. Therefore, a qRT-PCR analysis was conducted to study the dynamic expression patterns of all *PbCOBRA* family members, and the timeline was redesigned as 1, 6, 12, 24 h given that the perception and early response to abiotic stresses predominantly occur at the initial stage of treatment.

**Figure 8:**
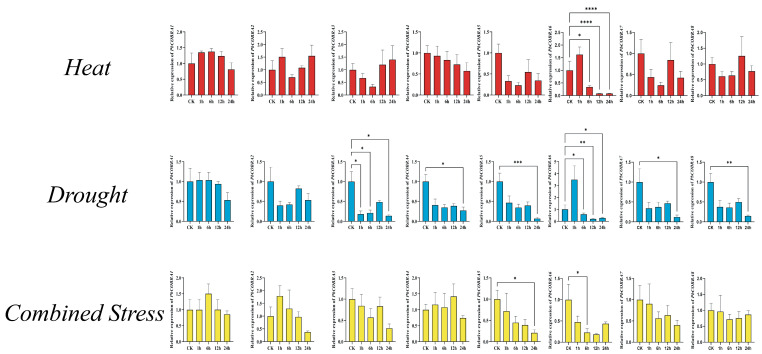
Expression Pattern Analysis of *PbCOBRA* Genes in *P. bournei* Under High Temperature, Drought, and Combined Heat-Drought Stress. Taking eight members of the *PbCOBRA* gene family (*PbCOBRA1*–*PbCOBRA8*) in *P. bournei* as the research objects, their relative expression levels at different time points (CK, 1 h, 6 h, 12 h, 24 h) under high temperature stress (Heat), drought stress (Drought), and combined heat-drought stress (Combined Stress) were detected by qRT-PCR. The data are presented as the mean ± standard error (SE) of three biological replicates; asterisks (*, **, ***, ****) represent significant differences between the treatment groups and the control group at *p* < 0.05, *p* < 0.01, *p* < 0.001, and *p* < 0.0001, respectively.

The results were shown in Figure 8, which illustrated that *PbCOBRA* genes exhibited diverse trends and dynamic expression alterations under different stresses. Among all genes, *PbCOBRA6* exhibited a pattern of strong response to environmental pressures. Facing single heat and drought hardship, *PbCOBRA6* showed a similar response mechanism, with a peak in 1 h followed by a significant decrease. This unique pattern suggests that *PbCOBRA6* may be associated with early stress-response processes. Under combined stress, *PbCOBRA6* showed a different mode, with a drop in the early stage and a slight increase in 24 h. This suggests that *PbCOBRA6* may have a stage-dependent association with combined stress responses, particularly during the later phase of stress adjustment. Importantly, the induction of *PbCOBRA1*, *PbCOBRA2* and *PbCOBRA4* varied with stress type and occurred at different time points. To be specific, *PbCOBRA1* was induced in the early and middle period of heat stress (1–12 h) and displayed a peak at 6 h under combined stress. Yet, it remained unresponsive or downregulated under drought. Simultaneously, *PbCOBRA2* exhibited an earlier induction, peaking at 1 h under both heat and combined stresses. However, similar to *PbCOBRA1*, it remained at relatively low expression levels under pure drought, with only a fluctuation at 12 h. Conversely, the expression profile of *PbCOBRA4* suggested a potential association with combined stress responses. Although it was suppressed under single heat and drought, it was specifically induced during the combined stress treatment, peaking at 12 h. This behavior indicates that distinct *PbCOBRA* members may exhibit stress-type-specific expression responses. Furthermore, the remaining genes, including *PbCOBRA3*, *PbCOBRA5*, *PbCOBRA7*, and *PbCOBRA8*, experienced generalized repression under the three stress treatments. These genes showed a downward trend across drought and combined stress conditions, though *PbCOBRA3* and *8* showed delayed recoveries under late heat stress. The qRT-PCR analysis elucidated the expression trends of the entire *PbCOBRA* family under the three stress conditions.

## 4. Discussion

The continuous high temperatures and drought conditions can cause irreversible damage to the plants [45]. Since plants mainly sustain themselves and contribute to the entire ecological environment through photosynthesis, high temperature and drought could lead to strict stomatal closure that limits photosynthetic capacity, thereby suppressing the growth and reproductive success of high-value tree species, posing a threat to the ecosystem [46]. Facing the drastic environmental pressures, the complex physiological regulatory mechanisms are the key to plant stress resistance [47]. Consequently, elucidating the regulatory networks within plant bodies holds significant guiding importance for the conservation of plant species and the breeding of stress-resistant varieties [48]. However, this network is dynamic and complex, making it difficult to fully reveal adaptation of plants under stress responses from a single perspective, such as genomic analysis alone [49]. The novelty of this study lies in placing the *PbCOBRA* gene family within an integrated transcriptomic and stress-trait association framework under single and combined abiotic stresses. Rather than presenting only a genome-wide inventory of *COBRA* genes, we connected *PbCOBRA* members with tissue-specific expression, stress-associated co-expression modules, physiological stress indicators, qRT-PCR expression dynamics, functional enrichment results, and predicted structural features. This integrated strategy allowed us to identify several promising *PbCOBRA* candidates, particularly *PbCOBRA6* and *PbCOBRA8* in the combined-stress-associated turquoise module, for future studies of cell wall-mediated stress adaptation in *P. bournei*.

### 4.1. Conserved Evolutionary Mechanism of PbCOBRA Gene Family

The *COBRA* gene family encodes plant-specific glycosylphosphatidylinositol (GPI)-anchored proteins that play key roles in cellulose deposition and directed cell elongation. Evolutionary analysis reveals an absence of duplication events in the *PbCOBRA* gene family. Chromosomal mapping showed that (Figure 3), with the exception of a small cluster comprising *PbCOBRA1* and *PbCOBRA2* on Chr01, all other *PbCOBRA* genes were dispersed individually across different chromosomes. Consistent with this, no intraspecific collinearity was detected within the family. All the results suggested a conserved expansion mechanism in *PbCOBRA* gene family, which was likely constrained by purifying selection. As the first physical barrier of plant cells, the cell wall forms the fundamental basis for normal cellular activities, which underscores the prominent importance of the *COBRA* gene family [50]. Thus, the functions carried by this family are so essential that may have constrained its duplication events, preventing mutations and functional redundancy from imposing severe fitness costs on the plant. This conserved evolutionary strategy also aligns with studies in the *COBRA* gene family of diverse plant species [26]. Despite this evolutionary conservation, abundant evidence points toward functional diversification among the eight *PbCOBRA* members. According to the subcellular localization prediction, PbCOBRA2 was the only protein predicted to localize to the vacuole, hinting at functions that might differ from those of plasma membrane-localized members. This divergence is further supported by the WGCNA results, which assigned four members to three distinct modules. Even though two *PbCOBRA* members (*PbCOBRA6* and *PbCOBRA8*) were co-assigned to the same module, their kME values were opposite (kME = 0.41 and −0.51, respectively), suggesting their distinctive roles in the module. The other two members severally belonged to red and black modules, which appeared to handle different stress challenges. Based on Figure 5, the red and black modules were associated with drought and heat responses, respectively. The relatively high kME values of PbCOBRA1 and PbCOBRA4 indicate strong module membership, supporting their potential importance within the corresponding stress-associated co-expression modules. These findings all indicated that individual *PbCOBRA* members likely fulfill distinct roles across diverse physiological processes in *P. bournei*, and thus represent key candidates for future investigation.

### 4.2. Structural Features and Putative Conceptual Model of PbCOBRA Proteins

Through the three-dimensional modeling of PbCOBRA proteins, highly similar structural features were discovered. This might indicate that though there may be specific functional differentiation among the members, their functions in cellulose deposition and directional elongation are highly conserved. All proteins have a central β-barrel core with high confidence, while the two sides consist of intrinsically disordered N-terminal and C-terminal regions, which are always considered as flexible areas [51]. The high-confidence regions may possess the core function of the PbCOBRA protein, which contain multiple N-glycosylation sites. Since N-glycosylation can enhance protein stability under stress and thereby improve stress tolerance, the abundant glycosylation sites in the key areas may protect PbCOBRA proteins and allow them to function stably under stress conditions [21,52]. Meanwhile, these glycosylation sites are all located in the rigid core, not in the flexible ends, suggesting the key protection of the cell for the core function of this protein. This may suggest a potential contribution of PbCOBRA proteins to stress-responsive processes in *P. bournei*. The significant enrichment of the “glycan biosynthesis and metabolism” pathway in the turquoise module under stress further supported this. This finding may reflect that plants actively synthesize sugar chains and glycosylate functional proteins to improve stress tolerance, and these four PbCOBRA proteins may be among the targets that the cell glycosylates [53,54]. In addition, the GPI anchor site is generally located in the flexible C-terminal region. Because GPI anchor sites are primarily responsible for anchoring proteins to the plasma membrane, this design potentially allows the proteins to maneuver freely within the complex cell wall matrix [18]. Under severe mechanical stress, this may help PbCOBRA proteins to remain firmly attached to the membrane while retaining enough freedom of movement to continuously guide cell wall remodeling. This protein architecture may support dynamic mechanical buffering and stable localization of PbCOBRA proteins at the plasma membrane–cell wall interface, providing a structural basis for their potential contribution under stress [20]. Based on the predicted three-dimensional architecture of PbCOBRA proteins, including the conserved core region, predicted N-glycosylation sites, flexible terminal regions, and C-terminal GPI-anchor site, we proposed a putative conceptual model for their possible roles at the plasma membrane–cell wall interface (Supplementary Figure S1). In this model, the GPI-anchor signal may tether PbCOBRA proteins to the outer surface of the plasma membrane, whereas the conserved core region may help maintain protein conformation. The predicted N-glycosylation sites may contribute to protein stability or extracellular function, and the flexible terminal regions may provide structural flexibility for positioning or interaction at the membrane–cell wall interface [21]. It should be emphasized that this model is hypothetical and is not presented as direct experimental evidence for PbCOBRA function. Rather, it integrates the structural predictions from this study with the stress-associated expression patterns, WGCNA results, and previously reported roles of COBRA/COBL proteins in cellulose deposition and cell wall organization. Future studies involving subcellular localization, protein modification validation, cell wall association assays, and functional genetic analyses will be required to verify this proposed model in *P. bournei*. A schematic illustration of this proposed conceptual framework is provided in the Supplementary Materials (Figure S1) [55].

### 4.3. Complex Stress Response Mechanisms in P. bournei

The results of WGCNA revealed that the plant response to combined stress is not a simple superposition of individual stress responses, reflecting the high complexity of biological systems. Notably, the turquoise module responded exclusively to combined stress with positive correlations with antioxidant capacities (SOD, CAT) and soluble protein content (SP), and it contains the most genes (5364 genes), highlighting its potential involvement in combined stress tolerance. This also indicates that plants may mobilize a broader and more diverse transcriptional program under combined stress. For example, *PbCOBRA6* and *PbCOBRA8* were co-assigned to the turquoise module with opposite kME values, suggesting their spatiotemporal functional divergence. The RNA-seq heatmap (Figure 5D) revealed that *PbCOBRA6* maintained a relatively high overall transcript abundance in the RNA pool, while that of *PbCOBRA8* was generally low. This contrasting pattern, together with their opposite kME values, may reflect the distinct involvement of these two members in stress adaptation. Moreover, there are huge differences in the cis-acting elements of the two genes. *PbCOBRA8* uniquely contains only environmental stress response elements, while *PbCOBRA6* possesses four different kinds of elements, including growth elements, environmental stress responsive elements, light response elements, and hormone response elements. This provides evidence for the functional differences between the two genes, and the limited elements may restrict the participation of *PbCOBRA8* in diverse pathways. Furthermore, they showed distinct subcellular localizations. PbCOBRA6 was predicted to localize to the plasma membrane, and PbCOBRA8 was predicted to localize to the chloroplast. Combining the functional enrichment results (Figure 6) with qRT-PCR analysis (Figure 8), it was hypothesized that they might be involved in different biological processes. Specifically, the early suppression of *PbCOBRA6* might reflect the temporary disruption caused by acute stress shock. Then, the slight recovery at 24 h may suggest its involvement in the later stage of the stress response. By contrast, plant chloroplasts can be highly vulnerable under extreme combined stress, and the related functions could be globally inhibited to prevent severe oxidative damage. Therefore, the persistently low expression and negative kME value of *PbCOBRA8* likely reflect this global suppression rather than an active regulatory response. Although the specific functions of the two genes need to be further verified through experiments, this subtle difference exemplifies that plant stress tolerance is realized by precisely coordinated and large-scale networks rather than by isolated pathways, highlighting the importance of a comprehensive understanding of stress responses [16,56,57]. Since plants typically grow in complex and fluctuating natural environments, they are rarely exposed to a single stress. Instead, multiple stresses often act simultaneously, collectively threatening plant growth and reproduction [58,59,60]. Although recent studies have still primarily focused on the response mechanisms of individual pressures [61,62], our findings emphasize the necessity of multi-stress research. [63].

Overall, this study is the first to conduct an in-depth exploration of the *COBRA* gene family of *P. bournei*. And by combining transcriptome and genome analysis, it reveals the potential roles of its members under single and combined stress conditions. Although some of the conclusions and models still require further experimental verification, this study provides potential genetic resources and theoretical basis for the breeding of drought-resistant *P. bournei*.

## 5. Conclusions

In summary, this study conducted a systematic analysis of the *COBRA* gene family in *P. bournei*, revealing its evolutionary conservation, tissue specificity, dynamic responses to stress, and integration into co-expression networks. The main conclusion of this study is that *PbCOBRA* genes are promising candidates potentially associated with cell wall-mediated responses to heat, drought, and combined heat–drought stress in *P. bournei*. The results showed that *PbCOBRA1* is a candidate gene potentially associated with xylem secondary cell wall formation, while *PbCOBRA6* showed an early-responsive expression pattern under high temperature and drought conditions. Notably, the turquoise module specifically responded to combined stress, and was significantly enriched in cell wall polysaccharide metabolism and peroxisome pathways. Two genes, *PbCOBRA6* and *PbCOBRA8*, were assigned to this module with opposite kME signs, indicating distinct co-expression relationships with the turquoise module eigengene. Furthermore, protein structure prediction supports the essential roles of N-glycosylation and GPI anchoring in maintaining structural stability and dynamic attachment at the cell wall–plasma membrane interface, leading to the proposal of a novel working model based on these findings. These findings provide new insights into the potential involvement of *PbCOBRA* genes in cell wall-mediated responses to combined climate stress in woody plants and lay a theoretical foundation for subsequent functional studies and stress-resistance breeding in *P. bournei*.

## Figures and Tables

**Table 1:** Primer sequences of quantitative RT-qPCR.

Primer	Sequence (5′~3′)
PbEF1α_1_F	GCTCCTGGTCACCGTGAC
PbEF1α_1_R	TCACGAGTTTGCCCGTCC
PbCBP20-F	GAAGGCAGACAATGGG
PbCBP20-R	ATAGTGCGATGGGACAG
PbCOBRA1-F	CAGCCTTCCAGGTGAGTGTT
PbCOBRA1-R	GTGAAGAAGGTGGTGGAGGG
PbCOBRA2-F	ATCTGCTCATGCAAGCTGGT
PbCOBRA2-R	CCGAGGAAAAGCCCATCCTT
PbCOBRA3-F	TGAGGAAGGATCCGGGCATA
PbCOBRA3-R	GTATCTGGTGGGGGCATCAC
PbCOBRA4-F	GTCGGTGACGACGGAGATAC
PbCOBRA4-R	GCAAAGGCATCTCGCAGTTC
PbCOBRA5-F	CGCATGCATTGCTTCTTCCA
PbCOBRA5-R	ATCGCCACATGGTAATCGCT
PbCOBRA6-F	AACCCATTGCCTTGTGGTGA
PbCOBRA6-R	GCACAGCAGCAAACCAATCA
PbCOBRA7-F	CAATGGAACAAGGATGCCGC
PbCOBRA7-R	GATCGATGGATGGGTGGGTC
PbCOBRA8-F	AATTGATGTGGCTGGAGGGG
PbCOBRA8-R	TCTGTATGCATGCCCTGTGG

**Table 2:** Physicochemical properties of COBRAs in *P. bournei*.

Gene Name	Gene ID	Number of Amino Acid	Molecular Weight/kDa	Theoretical pI	Instability Index	Aliphatic Index	GRAVY	Subcellular Localization
*PbCOBRA1*	*OF19810*	464	52.008	9.12	31.49	73.32	−0.193	Plasma membrane
*PbCOBRA2*	*OF19809*	457	50.970	8.74	29.17	75.27	−0.063	Vacuole
*PbCOBRA3*	*OF25674*	411	46.379	8.17	44.11	68.52	−0.21	Cytoplasm
*PbCOBRA4*	*OF05174*	438	48.448	5.5	37.54	80.55	−0.131	Endoplasmic reticulum
*PbCOBRA5*	*OF29927*	661	73.773	8.65	31.79	71.54	−0.331	Plasma membrane
*PbCOBRA6*	*OF06024*	648	70.482	5.71	34.43	75.91	−0.058	Plasma membrane
*PbCOBRA7*	*OF08601*	650	72.580	7.75	39.46	73.09	−0.307	Plasma membrane
*PbCOBRA8*	*OF00672*	544	59.708	6.15	44.67	79.82	−0.053	Chloroplast

## Data Availability

The original contributions presented in this study are included in the article/Supplementary Materials. Further inquiries can be directed to the corresponding authors.

## Supplementary Materials

The following supporting information can be downloaded at: https://www.mdpi.com/article/10.3390/biology15131084/s1. Figure S1: Putative conceptual model of PbCOBRA proteins at the plasma membrane–cell wall interface; Table S1: Adjusted_P_values; File S1: Alignment.

## Abbreviations

The following abbreviations are used in this manuscript:

CNGB	China National GeneBank
RGAP	Rice Genome Annotation Project
