# Supplementary material for: Integrated Genomic and Transcriptomic Analyses Suggest the Potential Involvement of the COBRA Gene Family in Heat, Drought and Combined Stress Responses in Phoebe bournei
Source: Biology (Basel). 2026 Jul 6;15(13):1084. doi: 10.3390/biology15131084 (PMC13359968; doi:10.3390/biology15131084)
Supplement: Supplementary file 1 [file biology-15-01084-s001.zip › Supplementary_Materials.pdf]

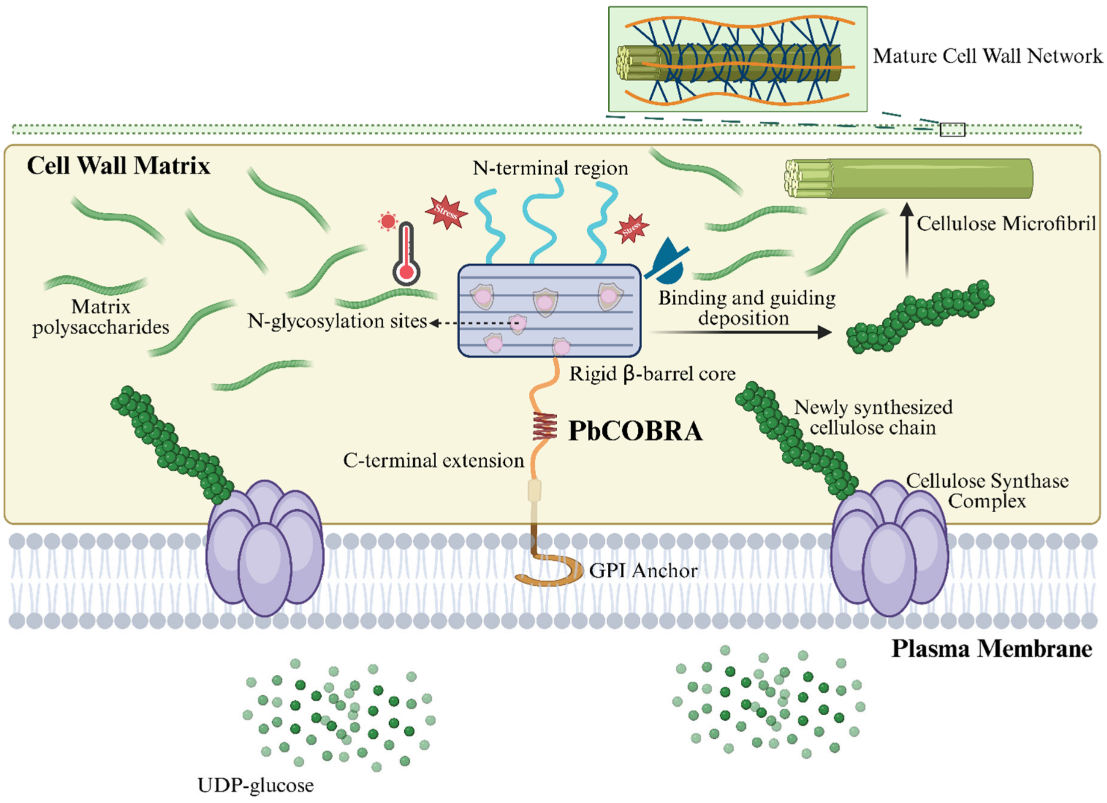

Figure S1. Putative conceptual model of PbCOBRA proteins at the plasma membrane–cell wall interface.
